# Efficacy of 11 anticoagulants for the prevention of venous thromboembolism after total hip or knee arthroplasty: A systematic review and network meta-analysis

**DOI:** 10.1097/MD.0000000000032635

**Published:** 2023-01-13

**Authors:** Zhihao Huang, Xinru Xu, Dan Xu, Pengfei Zhao, Miao Zou

**Affiliations:** a School of Big Data and Fundamental Sciences, Shandong Institute of Petroleum and Chemical Technology, Dongying, China; b College of Food Science, Northeast Agricultural University, Harbin, China; c Obstetrical department, Lijin County Central Hospital, Dongying, China; d Department of Clinical Pharmacy, Weifang People’s Hospital, Weifang, China.

**Keywords:** anticoagulants, deep vein thrombosis (DVT), network meta-analysis, pulmonary embolism (PE), total hip arthroplasty, total knee arthroplasty (TKA), venous thromboembolism (VTE)

## Abstract

To systematically review the efficacy of 11 anticoagulants in the treatment of venous thromboembolism (VTE) after total hip or knee arthroplasty.

**Methods:**

PubMed, Cochrane Library, Embase, Web of Science, China National Knowledge Infrastructure, Wanfang Data, VIP, and China Biology Medicine databases were electronically searched for studies assessing the efficacy of different anticoagulants for the prevention of VTE after total hip or knee arthroplasty from January 1, 2010, to January 27, 2022. Two reviewers independently screened the literature, extracted data, and graded the evidence using Confidence in Network Meta-Analysis. The network meta-analysis was then performed using Stata 16.0 software and R 4.1.0 software.

**Results:**

A total of 61 articles were included. The results of network meta-analysis showed that apixaban, edoxaban, fondaparinux, rivaroxaban, and darexaban were the most effective anticoagulants for the prevention of deep vein thrombosis in patients undergoing total hip or knee arthroplasty (*P* < .05), while there was no difference in the efficacy among the anticoagulants for the prevention of pulmonary embolism (*P* > .05).

**Conclusion:**

Apixaban, edoxaban, fondaparinux, rivaroxaban, and darexaban have the best efficacy for the prevention of VTE after total hip or knee arthroplasty.

## 1. Introduction

Venous thromboembolism (VTE), which comprises deep vein thrombosis (DVT) and pulmonary embolism (PE),^[1]^ with 10 million cases occurring every year,^[2]^ represents the third most common acute cardiovascular syndrome (after myocardial infarction and stroke),^[3]^ causes significant morbidity and mortality,^[4]^ and places a substantial clinical and economic burden.^[5]^ Total hip arthroplasty (THA) or total knee arthroplasty (TKA) is generally regarded as a highly successful surgical intervention that relieves pain, improves function, and enhances the quality of patients’ lives. However, VTE represents a major complication of this type of surgery. Among various methods of prophylaxis, the main approach involves anticoagulant prophylaxis. In addition, there are many kinds of anticoagulants, and there is no exact comparison of their anticoagulant effects. Therefore, in this study, we explored the effect of different anticoagulants on VTE prevention after THA or TKA through network meta-analysis, which is of great significance to guide clinical medical personnel to use scientific research results to prevent VTE.

## 2. Materials and methods

This review adhered to the Preferred Reporting in Systematic Reviews and Meta-Analysis 2020 guidelines,^[6]^ and this review was registered in the International Prospective Register of Systematic Reviews (registration number, CRD42022357393).

## 3. Search strategy

A literature search was carried out by 2 independent reviewers. PubMed, Embase, The Cochrane Library, Web of Science, CBM, CNKI, WanFang Data, and VIP were explored from January 1, 2000, to January 27, 2022. To minimize the missing literature, references listed in the included studies were also traced to supplement relevant data.

## 4. Eligibility criteria

The inclusion criteria were as follows: administration of anticoagulants after THA or TKA (not limited by age, race, and nationality of patients); prospective or retrospective study design; direct or indirect availability of the results – responders and sample size.

The exclusion criteria were as follows: duplicate articles; articles with inconsistent research contents; review articles; conference abstracts; animal studies; case reports; study protocols; and non-English and non-Chinese articles.

### 4.1. Literature screening and data extraction

Two reviewers independently screened the literature and extracted and cross-checked the data. In case of disagreements, a third party was consulted to assist in the judgment. During literature screening, first, the title and abstract were read. Then, after the exclusion of irrelevant literature, the full text of the preliminarily relevant articles was read to determine whether to include them in the final analysis. Data extraction encompassed the basic characteristics of the included studies, such as author, publication year, country, anticoagulant, age, recipients, mean body mass index, duration of surgery, and surgery type. Results considered responders and sample size.

### 4.2. Statistical analysis

“Network” package of Stata 16.0 software was used to produce the network plot. The size of the nodes corresponds to the number of participants randomized to each treatment. Treatments with direct comparisons are linked with a line; its thickness corresponds to the number of trials evaluating the comparison. Frequentist network meta-analysis was conducted using the “netmeta” package in R 4.1.0 software. Transitivity was subjectively assessed by the basic characteristics of the included studies. *I*^2^ was used to analyze the heterogeneity. If *I*^2^ < 50%, there was little heterogeneity between the studies, and the fixed-effects model was used for pooling. If *I*^2^ ≥ 50%, there was great heterogeneity between the studies. Meta-regression was used to identify the potential factors causing heterogeneity, and then subgroup analysis was performed. If the source of heterogeneity could not be found, the random-effects model was used for pooling. Inconsistency was divided into global inconsistency and local inconsistency. Global Wald test was used to evaluate global inconsistency, and the node-splitting test was used to evaluate local inconsistency. If there was no statistically significant difference between the results of direct comparison and indirect comparison (*P* > .05), the consistency was good, and the consistency model was used for pooling; otherwise, the inconsistent model was used. Relative risk (RR) and 95% confidence interval (CI) were used to evaluate the efficacy of anticoagulants in the league table. *P* score was used to rank and compare different anticoagulants. The higher the *P* score value, the higher the efficacy ranking of the anticoagulant, and vice versa. The stability of the research results was analyzed by sensitivity analysis. The included studies were excluded one by one, and then meta-analysis was performed again. The results were compared with those before exclusion. If the change was small, it indicated that the stability of the included literature was good and the results were credible. If there were significant changes, it indicated that the results were not credible. A funnel plot was used to evaluate the existence of publication bias. If the *P* values of Egger, Begg–Mazumdar, and Thompson–Sharp tests were >.05, there was no publication bias; otherwise, there was publication bias.

### 4.3. Evidence assessment of the included studies

The reviewers assessed the certainty of evidence contributing to network estimates of the main outcomes using the Confidence in Network Meta-Analysis (CINeMA) framework,^[7]^ which includes 6 domains: within-study bias, reporting bias, indirectness, imprecision, heterogeneity, and incoherence. Within-study bias was assessed by the modified Jadad score.^[8]^ Potential sources of bias include random sequence production, allocation concealment, blinding method, and withdrawals and dropouts. According to the modified Jadad score, studies with a score ≤2 were considered studies without concerns; studies with a score 3 to 5 were considered studies with some concerns; and studies with a score ≥6 were considered studies with major concerns. Reporting bias was assessed subjectively in accordance with unpublished studies, outcomes in the gray literature, and funnel plots. Indirectness was subjectively assessed by the basic characteristics of the included studies. For imprecision, heterogeneity, and incoherence, relative effect estimates <0.800 and >1.250 were considered clinically important.

## 5. Results

### 5.1. Literature search and characteristics of the included studies

**Figure 1.**
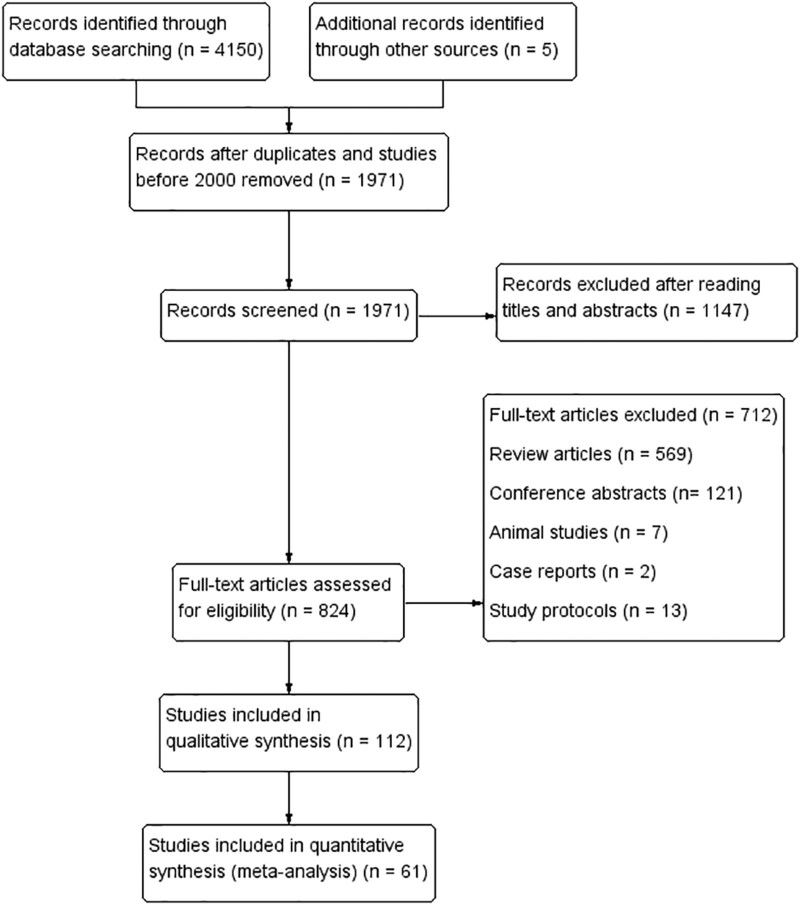
Flow diagram of the literature search and selection processes.

Sixteen anticoagulants were searched preliminarily by referring to relevant literature, and a total of 4150 articles were identified by searching the databases. Additional 5 articles were identified during the screening of the reference sections of the included articles. The detailed information is shown in Supplemental Method S1, Supplemental Digital Content, http://links.lww.com/MD/I301. After the layer-by-layer screening, 61 articles^[9–69]^ on 11 anticoagulants were finally included. The process and the results of the literature screening are shown in Figure 1. Detailed information on the included studies is shown in Table 1. The 11 anticoagulants included were apixaban, aspirin, betrixaban, dabigatran, darexaban, edoxaban, fondaparinux, low-molecular-weight heparin (LMWH), rivaroxaban, unfractionated heparin (UFH), and warfarin.

**Table 1:** Characteristics of the included studies.

No.	Study	Country	Anticoagulant	Age	Recipients (m/f)	Mean body mass index (SD), kg/m^2^	Duration of surgery, h	Surgery type
1	Anderson 2013	Canada	LMWH	57.9 ± 12.2	400 (213/187)	27.9 ± 5.8	1.53 ± 0.82	THA
			Aspirin	57.6 ± 11.9	385 (231/154)	29.3 ± 5.9	1.54 ± 0.62	
2	Anderson 2018	Canada	Rivaroxaban	60.9 ± 11.0	902 (480/422)	29.4 ± 5.8	1.4 ± 0.6	THA
			Aspirin	61.3 ± 11.1	902 (486/416)	29.4 ± 6.0	1.4 ± 0.6	
3	Anderson 2018	Canada	Rivaroxaban	64.7 ± 8.4	815 (353/462)	32.7 ± 6.8	1.4 ± 0.5	TKA
			Aspirin	64.6 ± 8.7	805 (318/487)	33.0 ± 7.2	1.4 ± 0.5	
4	Argun 2013	Turkey	Fondaparinux	58.7 ± 13.6	55 (21/34)	NA	NA	THA & TKA
			LMWH	60.0 ± 8.4	53 (20/33)	NA	NA	
5	Bai 2018	China	Rivaroxaban	69.28 ± 10.42	98 (50/48)	23.12 ± 3.29	NA	THA
			LMWH	68.33 ± 11.84	98 (49/49)	22.85 ± 2.85	NA	
6	Bai 2020	China	Rivaroxaban	70 ± 7	42 (4/38)	27 ± 3	NA	TKA
			LMWH	71 ± 8	42 (7/35)	27 ± 4	NA	
7	Bai 2021	China	Rivaroxaban	61.2 ± 11.7 (27–83)	114 (42/72)	25.7 ± 3.2 (20.1–29.7)	NA	THA
			LMWH	61.9 ± 9.6 (32–82)	114 (51/63)	24.6 ± 3.1 (21.2–29.2)	NA	
8	Bauer 2001	USA	Fondaparinux	67.5 ± 10.7	517 (204/313)	31.5 ± 6.5	2.12 ± 0.65	TKA
			LMWH	67.5 ± 10.2	517 (223/294)	30.9 ± 6.2	2.13 ± 0.7	
9	Bonneux 2006	Belgium	Fondaparinux	66.9 ± 8.5	55 (12/43)	29.7 ± 5.2	NA	TKA
			LMWH	65.7 ± 10.4	54 (11/43)	29.8 ± 7.8	NA	
10	Colleoni 2008	Brazil	Aspirin	71.21 ± 6.35	14 (1/13)	NA	NA	TKA
			Rivaroxaban	67.11 ± 7.65	18 (4/14)	NA	NA	
11	Ding 2014	China	Rivaroxaban	56.5 ± 18.2 (35–72)	120 (78/42)	NA	NA	THA
			LMWH			NA	NA	
12	Eriksson 2005	Sweden	Dabigatran	65.9 (33–93)	393 (164/229)	NA	1.4 (0.5–3.6)	THA & TKA
			LMWH	65.0 (20–86)	392 (151/241)	NA	1.5 (0.4–4.6)	
13	Eriksson 2006	Sweden	Rivaroxaban	67 (51–87)	37 (16/21)	28 (21–38)	1.53 ± 0.55	THA
			LMWH	65 (27–82)	132 (54/78)	28 (20–40)	1.37 ± 0.48	
14	Eriksson 2007	Sweden	Dabigatran	67 ± 9	679 (238/441)	NA	1.52 ± 0.47	TKA
			LMWH	68 ± 9	694 (216/478)	NA	1.5 ± 0.47	
15	Eriksson 2007	Sweden	Dabigatran	67 ± 9	679 (238/441)	NA	1.52 ± 0.47	TKA
			LMWH	68 ± 9	694 (216/478)	NA	1.5 ± 0.47	
16	Eriksson 2007	Sweden	Dabigatran	65 ± 10	1146 (510/636)	NA	1.42 ± 0.48	THA
			LMWH	64 ± 11	1154 (503/651)	NA	1.45 ± 0.48	
17	Eriksson 2007	Sweden	Dabigatran	65 ± 10	1146 (510/636)	NA	1.42 ± 0.48	THA
			LMWH	64 ± 11	1154 (503/651)	NA	1.45 ± 0.48	
18	Eriksson 2007	Sweden	Rivaroxaban	66 (32–84)	77 (32/45)	28 (18–38)	NA	THA
			LMWH	64 (30–92)	162 (74/88)	28 (19–44)	NA	
19	Eriksson 2007	Sweden	Darexaban	NA	NA	NA	NA	THA
			LMWH	NA	NA	NA	NA	
20	Eriksson 2008	Sweden	Rivaroxaban	63.1 (18–91)	2209 (989/1220)	27.8 (16.2–53.4)	1.51 (0.45–8.00)	THA
			LMWH	63.3 (18–93)	2224 (982/1242)	27.9 (15.2–50.2)	1.52 (0.42–5.75)	
21	Eriksson 2010	Sweden	Darexaban	61.3 (24–84)	163 (73/90)	28.5 (18.3–40.8)	1.39 (0.4–4.0)	THA
			LMWH	58.1 (22–85)	166 (80/86)	27.3 (18.4–41.4)	1.50 (0.5–3.4)	
22	Eriksson 2011	Sweden	Dabigatran	62 ± 12	1010 (469/541)	27.8 ± 4.8	1.33 (0.25–5.50)	THA
			LMWH	62 ± 11	1003 (502/501)	27.8 ± 4.8	1.32 (0.47–4.00)	
23	Eriksson 2011	Sweden	Dabigatran	62 ± 12	1010 (469/541)	27.8 ± 4.8	1.33 (0.25–5.50)	THA
			LMWH	62 ± 11	1003 (502/501)	27.8 ± 4.8	1.32 (0.47–4.00)	
24	Fizgerald 2001	USA	Warfarin	NA	349 (153/196)	NA	NA	THA
			LMWH	NA		NA	NA	
25	Fuji 2014	Japan	Edoxaban	72,6 ± 7.5 (36–84)	299 (54/245)	NA	1.85 ± 0.63 (0.52–3.75)	TKA
			LMWH	72.1 ± 7.8 (30–84)	295 (66/229)	NA	1.90 ± 0.53 (0.55–3.73)	
26	Fuji 2014	Japan	Darexaban	62.1 ± 10.48	136 (23/113)	23.92 ± 3.168	1.79 ± 0.702	THA
			LMWH	61.6 ± 10.99	82 (20/62)	23.70 ± 3.704	1.68 ± 0.710	
27	Fuji 2014	Japan	Darexaban	71.2 ± 7.88	71 (9/62)	26.36 ± 3.850	1.82 ± 0.636	TKA
			LMWH	72.3 ± 8.02	66 (9/57)	26.43 ± 3.407	1.80 ± 0.503	
28	Fuji 2015	Japan	Edoxaban	62.8 ± 9.61	255 (35/220)	24.5 ± 3.52		THA
			LMWH	62.8 ± 9.72	248 (36/212)	24.2 ± 3.60		
29	Gao 2011	China	LMWH	66.1 (22–82)	166 (27/139)	26.79 ± 3.87		TKA
			Aspirin	64.9 (40–84)	120 (21/99)	27.87 ± 3.62		
30	Gao 2016	China	LMWH	59.2 ± 7.7 (36–74)	54 (30/24)	23.6 ± 4.8		THA
			Rivaroxaban	59.5 ± 7.8 (35–78)	54 (31/23)	23.4 ± 4.5		
31	Ginsberg 2009	Canada	Dabigatran	66.2 ± 9.5	857 (371/486)	NA	1.52 ± 0.47	TKA
			LMWH	66.3 ± 9.6	868 (364/504)	NA	1.50 ± 0.47	
32	Guo 2018	China	LMWH	63.6 ± 2.5 (52–82)	60 (27/33)	NA	NA	THA & TKA
			Rivaroxaban			NA	NA	
33	Hass 2006	Germany	LMWH	66.1 ± 9.3	1013 (337/676)	27.8 ± 3.8	1.42 (0.50–5.33)	THA & TKA
			UFH	66.9 ± 9.8	1005 (350/655)	27.8 ± 3.9	1.42 (0.47–4.33)	
34	Hosaka 2013	Japan	Fondaparinux	73.3 ± 7.3	277 (31/246)	26.4 ± 3.9	1.89 ± 0.51	TKA
			LMWH	72.8 ± 7.7	298 (31/267)	26.4 ± 5.2	1.89 ± 0.48	
35	Hull 2000	Canada	LMWH	64 ± 13	983 (467/516)	29 ± 6		THA
			Warfarin	63 ± 13	489 (242/247)	28 ± 5		
36	Jiang 2019	China	Apixaban	68.7 ± 5.7	110 (62/48)	24.5 ± 3.2	1.84 ± 0.39	TKA
			LMWH	70.2 ± 6.1	110 (52/58)	25.1 ± 3.5	1.76 ± 0.33	
37	Kakkar 2000	UK	LMWH	70.4 ± 10.9	149 (49/100)	25.3 ± 4.1	1.83 ± 0.92	THA
			UFH	70.5 ± 9.2	149 (45/104)	25.6 ± 4.6	1.68 ± 0.98	
38	Kakkar 2008	UK	Rivaroxaban	61.4 ± 13.2 (18–93)	1228 (561/667)	26.8 ± 4.8 (15.6–54.7)	1.58 (0.50–7.92)	THA
			LMWH	61.6 ± 13.7 (19–93)	1229 (578/651)	27.1 ± 5.2 (15.5–59.0)	1.55 (0.47–9.92)	
39	Kim 2016	South Korea	Rivaroxaban	55.9 ± 14.30	350 (163/187)	25.0 ± 3.20	1.22 ± 0.45	THA
			LMWH	56.0 ± 15.17	351 (174/177)	25.0 ± 3.59	1.25 ± 0.51	
40	Lassen 2002	Denmark	Fondaparinux	67 (30–90)	908 (396/512)	26 (15–45)	2·3 ± 0·80	THA
			LMWH	67 (24–97)	919 (402/517)	26 (14–51)	2.4 ± 0.87	
41	Lassen 2002	Denmark	Fondaparinux	66 (29–92)	1140 (493/647)	26 (15–45)	2.3 ± 0.82	THA
			LMWH	67 (24–97)	1133 (473/660)	27 (14–51)	2·4 ± 0·83	
42	Lassen 2007	Denmark	Apixaban	66.4 (46–84)	157 (54/103)	30.2 (22.5–50.2)	1.60 (0.62–7.73)	TKA
			LMWH	66.5 (36–88)	152 (58/94)	30.4 (18.8–46.0)	1.60 (0.70–3.33)	
			Warfarin	66.8 (43–85)	153 (60/93)	30.4 (20.8–50.1)	1.61 (0.67–4.17)	
43	Lassen 2008	Denmark	Rivaroxaban	67.6 (28–91)	1220 (363/857)	29.5 (16.3–51.1)	1.60 (0.43–8.33)	TKA
			LMWH	67.6 (30–90)	1239 (418/821)	29.8 (16.0–54.3)	1.62 (0.47–5.25)	
44	Li 2018	China	Rivaroxaban	NA (35–75)	50 (13/37)	NA	NA	THA & TKA
			LMWH	NA (36–75)	50 (21/29)	NA	NA	
45	Migita 2014	Japan	Fondaparinux	73.9 ± 8.0 (34–93)	1294 (221/1073)	25.4 ± 3.9 (14.5–44.0)	2.11 ± 0.62 (0.70–6.67)	TKA
			LMWH					
			UFH					
46	Migita 2014	Japan	Fondaparinux	66.7 ± 10.5 (23–94)	868 (128/740)	24.5 ± 3.9 (14.5–44.0)	2.06 ± 0.72 (0.58–5.53)	THA
			LMWH					
			UFH					
47	Mirdamadi 2014	Iran	LMWH	68.3 ± 10.1	45 (15/30)	NA	NA	TKA
			Dabigatran	72.1 ± 9.3	45 (17/28)	NA	NA	
48	Qin 2016	China	Rivaroxaban	60.5 ± 4.1 (36–79)	50 (29/21)	NA	NA	THA
			LMWH	61.1 ± 4.2 (37–78)	50 (28/22)	NA	NA	
49	Quan 2010	China	Rivaroxaban	63.9 ± 14.9	48 (13/35)	23.72 ± 2.61	NA	THA & TKA
			LMWH	57.2 ± 16.9	36 (10/26)	24.50 ± 2.07	NA	
50	Rahman 2020	Egypt	Rivaroxaban	42.95 ± 10.6	80 (36/44)	30.5 ± 4.80	1.67 ± 0.12	THA
			LMWH	40.10 ± 14.7	80 (44/36)	29.8 ± 4.05	1.69 ± 0.13	
51	Raskob 2010	USA	LMWH	57.6 ± 12.41	175 (68/107)	27.10 ± 4.27	1.36 ± 0.45	THA
			Edoxaban	58.3 ± 11.55	187 (68/119)	28.53 ± 4.82	1.39 ± 0.45	
52	Ren 2021	China	Aspirin	54.5 (40.8–62.3)	34 (13/21)	23.6 (20.7–25.5)	NA	THA
			Rivaroxaban	50.0 (36.8–57.0)	36 (11/25)	23.5 (20.3–26.0)	NA	
53	Senaran 2005	Turkey	LMWH	55.2 ± 8.4	50 (12/38)	NA	NA	THA
			UFH	52.4 ± 11.2	50 (17/33)	NA	NA	
54	Shi 2014	China	Rivaroxaban	65.14 ± 8.93	50 (10/40)	26.53 ± 3.56	NA	TKA
			LMWH	66.84 ± 6.90	25 (7/18)	27.19 ± 3.71	NA	
55	Turpie 2002	Canada	Fondaparinux	67 (26–92)	908 (386/401)	28 (14–73)	2.46 ± 0.95	THA
			LMWH	67 (19–91)	919 (375/422)	27 (13–83)	2.42 ± 0.98	
56	Turpie 2002	Canada	Fondaparinux	67 (18–92)	1128 (556/572)	28 (14–73)	2.48 ± 0.95	THA
			LMWH	67 (19–91)	1129 (522/607)	28 (13–83)	2.45 ± 0.95	
57	Turpie 2005	Canada	Rivaroxaban	67 (49–84)	103 (37/66)	31.8 ± 6.3	1.47 ± 0.57	TKA
			LMWH	66 (47–83)	104 (47/57)	31.8 ± 6.0	1.51 ± 0.49	
58	Turpie 2009	Canada	Rivaroxaban	64.4 ± 9.7	1526 (519/1007)	30.9 ± 6.2	1.67 ± 0.71	TKA
			LMWH	64.7 ± 9.7	1508 (541/967)	30.7 ± 6.0	1.67 ± 0.70	
59	Turpie 2009	Canada	Betrixaban	65 (47–75)	84 (32/52)	NA	NA	TKA
			LMWH	62 (43–75)	43(21/22)	NA	NA	
60	Wang 2014	China	Rivaroxaban	68.1 ± 0.5 (55–75)	60 (35/25)	NA	NA	TKA
			LMWH	67.5 ± 0.3 (57–73)	60 (36/24)	NA	NA	
61	Wang 2017	China	Rivaroxaban	69.3 ± 3.7	96 (27/69)	27.1 ± 4.4	1.43 ± 0.12	TKA
			LMWH	70.7 ± 4.5	99 (34/65)	28.6 ± 3.9	1.51 ± 0.12	
62	Wang 2020	China	Rivaroxaban	64.18 ± 8.56	89 (40/49)	23.13 ± 1.60	NA	THA
			LMWH	63.70 ± 7.38	89 (44/45)	22.84 ± 1.45	NA	
63	Weitz 2020	Canada	LMWH	67.0 ± 8.8	76 (21/55)	32.4 ± 5.5	1.42 (1.08–1.82)	TKA
			Apixaban	64.9 ± 8.4	83 (18/65)	32.6 ± 5.8	1.42 (1.25–1.75)	
64	Wu 2013	China	Rivaroxaban	72.1	64 (25/39)	NA	NA	THA
			LMWH	74.7	64 (28/36)	NA	NA	
65	Yang 2013	China	Rivaroxaban	57.64 ± 10.22	75 (40/35)	24.28 ± 4.59	NA	THA
			LMWH	59.51 ± 10.65	70 (36/34)	23.80 ± 4.41	NA	
66	Yokote 2011	Japan	Fondaparinux	63.0 ± 10.0	84 (14/70)	22.5 ± 4.8	NA	THA
			LMWH	64.0 ± 11.0	83 (16/67)	23.0 ± 3.3	NA	
67	Zhang 2017	China	LMWH	58.36 ± 9.64 (47–69)	45 (26/19)	NA	NA	THA
			Rivaroxaban	56.68 ± 9.37 (45–68)	45 (27/18)	NA	NA	
68	Zhang 2020	China	LMWH	57.44 ± 9.89	43 (18/25)	26.11 ± 4.53	NA	TKA
			Rivaroxaban	59.72 ± 8.11	43 (21/22)	24.79 ± 3.48	NA	
69	Zou 2014	China	Rivaroxaban	63.5 (50–82)	102 (32/70)	27.5 (18.0–39.5)	1.42 (1.32–1.45)	TKA
			LMWH	65.7 (54–80)	112 (20/92)	27.0 (20.3–37.0)	1.41 (1.35–1.45)	
			Aspirin	62.7 (47–79)	110 (28/82)	27.8 (17.8–40.0)	1.51 (1.33–1.57)	

LMWH = low molecular weight heparin, NA = , SD = standard deviation, THA = total hip arthroplasy, TKA = total knee arthroplasty, UFH = unfractionated heparin.

### 5.2. Network meta-analysis

**Figure 2.**
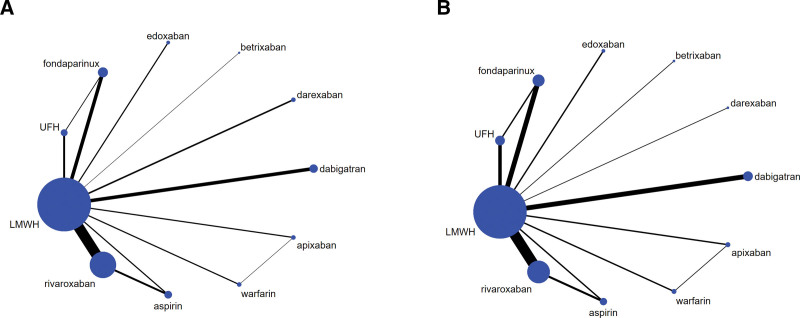
Network plots of overall efficacy. (A) DVT outcome. (B) PE outcome. The width of the lines is proportional to the number of trials comparing every pair of treatments, and the size of every node is proportional to the number of participants. DVT = deep vein thrombosis, PE = pulmonary embolism.

**Figure 3.**
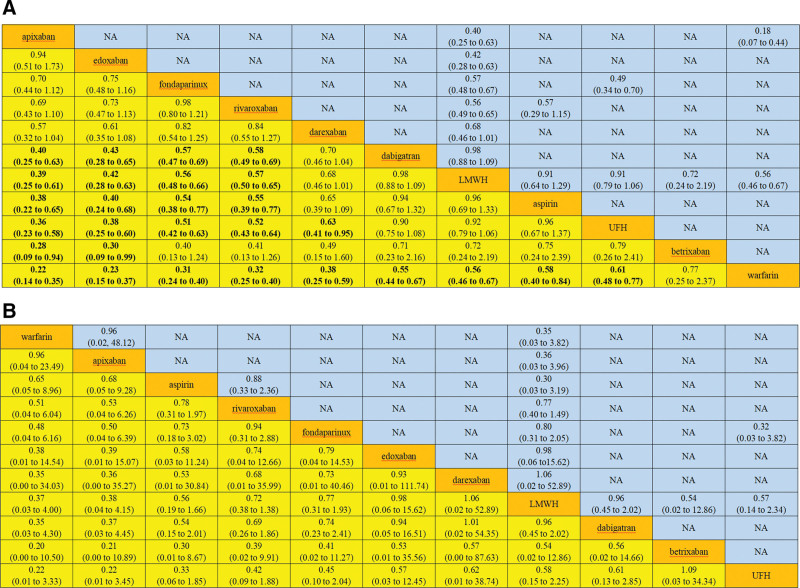
League tables of results. (A) DVT outcome. (B) PE outcome. Results of the network meta-analysis are presented in the left lower half and results from pairwise meta-analysis in the upper right half, if available. DVT = deep vein thrombosis, PE = pulmonary embolism.

**Figure 4.**
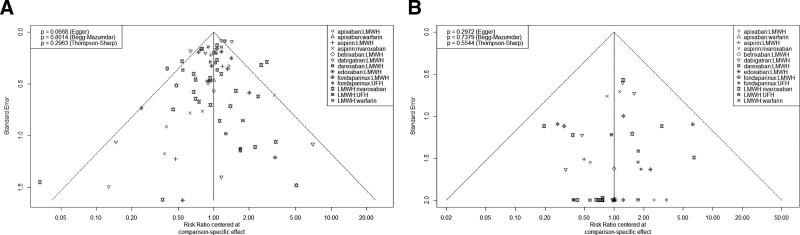
Funnel plots of results. (A) DVT outcome. (B) PE outcome. DVT = deep vein thrombosis, PE = pulmonary embolism.

To visualize network geometry and node connectivity, network plots were produced for each outcome (Fig. 2). There was a similarity after carefully reviewing the included studies. Hence, the assumption of transitivity was likely to hold in the data. There was no heterogeneity for DVT outcome (*I*^2^ = 43.9%) or PE outcome (*I*^2^ = 0.0%); no global inconsistency for DVT outcome (global Wald test: *P* = .675) or PE outcome (global Wald test: *P* = .960); and no local inconsistency for DVT outcome (Table 2) or PE outcome (Table 3). Therefore, the consistency model and fixed model were used for pooling. Sixty-one articles with 67 studies were included for DVT outcome (Table 4). In terms of prevention of DVT, efficacy of apixaban was better than that of dabigatran (RR = 0.40, 95% CI [0.25–0.63]), LMWH (RR = 0.39, 95% CI [0.25–0.61]), aspirin (RR = 0.38, 95% CI [0.22–0.65]), UFH (RR = 0.36, 95% CI [0.23–0.58]), betrixaban (RR = 0.28, 95% CI [0.09–0.94]), and warfarin (RR = 0.22, 95% CI [0.14–0.35]); efficacy of edoxaban was better than that of dabigatran (RR = 0.43, 95% CI [0.28–0.65]), LMWH (RR = 0.42, 95% CI [0.28–0.63]), aspirin (RR = 0.40, 95% CI [0.24–0.68]), UFH (RR = 0.38, 95% CI [0.25–0.60]), betrixaban (RR = 0.30, 95% CI [0.09–0.99]), and warfarin (RR = 0.23, 95% CI [0.15–0.37]); efficacy of fondaparinux was better than that of dabigatran (RR = 0.57, 95% CI [0.47–0.69]), LMWH (RR = 0.56, 95% CI [0.48–0.66]), aspirin (RR = 0.54, 95% CI [0.38–0.77]), UFH (RR = 0.51, 95% CI [0.42–0.63]), and warfarin (RR = 0.31, 95% CI [0.24–0.40]); efficacy of rivaroxaban was better than that of dabigatran (RR = 0.58, 95% CI [0.49–0.69]), LMWH (RR = 0.57, 95% CI [0.50–0.65]), aspirin (RR = 0.55, 95% CI [0.39–0.77]), UFH (RR = 0.52, 95% CI [0.43–0.64]), and warfarin (RR = 0.32, 95% CI [0.25–0.40]); efficacy of darexaban was better than that of UFH (RR = 0.63, 95% CI [0.41–0.95]) and warfarin (RR = 0.38, 95% CI [0.25–0.59]); efficacy of dabigatran was better than that of warfarin (RR = 0.55, 95% CI [0.44–0.67]); efficacy of LMWH was better than that of warfarin (RR = 0.56, 95% CI [0.46–0.67]); efficacy of aspirin was better than that of warfarin (RR = 0.58, 95% CI [0.40–0.84]); and efficacy of UFH was better than that of warfarin (RR = 0.61, 95% CI [0.48–0.77]) (Fig. 3A). The *P* score of the anticoagulants’ efficacy for the prevention of DVT was in the following order: apixaban > edoxaban > fondaparinux > rivaroxaban > darexaban > dabigatran > LMWH > aspirin > UFH > betrixaban > warfarin (Table 5). Thirty-nine articles with 42 studies were included in the analysis of PE outcome (Table 6). There was no significant difference in head-to-head comparisons of the efficacy of the 11 anticoagulants for the prevention of PE (Fig. 3B). The *P* score of the anticoagulants’ efficacy for the prevention of PE was in the following order: warfarin > apixaban > aspirin > rivaroxaban > fondaparinux > edoxaban > darexaban > LMWH > dabigatran > betrixaban > UFH (Table 7). After the exclusion of individual studies one by one, the remaining studies were pooled and analyzed again. The results showed that each excluded study had a minor impact on the amount of pooling effect, indicating that the results of this meta-analysis were stable and reliable. The results of funnel plots showed that there was no publication bias for the outcome of DVT (Egger test *P* = .067, Begg–Mazumdar test *P* = .801, Thompson–Sharp test *P* = .296) (Fig. 4A) or PE (Egger test *P* = .297, Begg–Mazumdar test *P* = .738, Thompson–Sharp test *P* = .554) (Fig. 4B).

**Table 2:** Result of node-splitting test for DVT.

Comparison	*P* value
LMWH vs apixaban	.676
Warfarin vs apixaban	.619
Aspirin vs LMWH	.443
Aspirin vs rivaroxaban	.880
Fondaparinux vs LMWH	.469
Fondaparinux vs UFH	.716
LMWH vs rivaroxaban	.174
LMWH vs UFH	.615
LMWH vs warfarin	.655

DVT = deep vein thrombosis, LMWH = low molecular weight heparin, UFH = unfractionated heparin.

**Table 3:** Result of node-splitting test for PE.

Comparison	*P* value
LMWH vs apixaban	.579
Warfarin vs apixaban	1.000
Aspirin vs LMWH	.557
Aspirin vs rivaroxaban	.449
Fondaparinux vs LMWH	.711
Fondaparinux vs UFH	.740
LMWH vs rivaroxaban	.424
LMWH vs UFH	.914
LMWH vs warfarin	.580

LMWH = low molecular weight heparin, PE = pulmonary embolism, UFH = unfractionated heparin.

**Table 4:** Characteristics of anticoagulants’ efficacy for prevention of DVT.

Study	Treatment	Responder	Sample size
1	LMWH	2	398
1	Aspirin	1	380
2	Rivaroxaban	3	902
2	Aspirin	2	902
3	Rivaroxaban	3	815
3	Aspirin	4	805
4	Fondaparinux	0	55
4	LMWH	1	53
5	Rivaroxaban	4	98
5	LMWH	11	98
6	Rivaroxaban	14	42
6	LMWH	10	42
7	Rivaroxaban	2	114
7	LMWH	4	114
8	Fondaparinux	45	361
8	LMWH	98	361
9	Fondaparinux	2	55
9	LMWH	1	54
10	Aspirin	1	14
10	Rivaroxaban	2	18
11	Rivaroxaban	5	60
11	LMWH	6	60
12	Dabigatran	39	297
12	LMWH	72	300
13	Rivaroxaban	2	29
13	LMWH	18	106
14	Dabigatran	181	503
14	LMWH	184	511
15	Dabigatran	1	675
15	LMWH	8	685
16	Dabigatran	40	874
16	LMWH	56	894
17	Dabigatran	6	1137
17	LMWH	1	1142
18	Rivaroxaban	6	59
18	LMWH	18	107
19	Darexaban	5	27
19	LMWH	12	31
20	Rivaroxaban	12	1595
20	LMWH	53	1558
21	Darexaban	16	120
21	LMWH	24	127
22	Dabigatran	60	791
22	LMWH	67	783
23	Dabigatran	0	1001
23	LMWH	4	992
24	Warfarin	72	122
24	LMWH	41	108
25	Edoxaban	22	299
25	LMWH	41	295
26	Darexaban	4	136
26	LMWH	2	82
27	Darexaban	11	71
27	LMWH	14	66
28	Edoxaban	6	255
28	LMWH	17	248
29	LMWH	37	166
29	Aspirin	28	120
30	LMWH	5	54
30	Rivaroxaban	4	54
31	Dabigatran	188	604
31	LMWH	163	643
32	LMWH	3	30
32	Rivaroxaban	1	30
33	LMWH	200	813
33	UFH	204	815
34	Fondaparinux	13	275
34	LMWH	18	296
35	LMWH	80	673
35	Warfarin	81	338
36	Apixaban	6	110
36	LMWH	22	110
37	Rivaroxaban	14	864
37	LMWH	71	869
38	LMWH	9	101
38	UFH	24	116
39	Rivaroxaban	24	350
39	LMWH	23	351
40	Fondaparinux	36	908
40	LMWH	83	918
41	Apixaban	5	105
41	LMWH	15	109
41	Warfarin	29	109
42	Rivaroxaban	79	824
42	LMWH	160	878
43	Rivaroxaban	0	50
43	LMWH	4	50
44	Fondaparinux	60	360
44	LMWH	59	223
44	UFH	24	72
45	Fondaparinux	17	261
45	LMWH	17	148
45	UFH	5	32
46	LMWH	1	45
46	Dabigatran	1	45
47	Rivaroxaban	3	50
47	LMWH	5	50
48	Rivaroxaban	10	48
48	LMWH	9	36
49	Rivaroxaban	8	80
49	LMWH	0	80
50	LMWH	20	144
50	Edoxaban	2	158
51	Aspirin	3	34
51	Rivaroxaban	3	36
52	LMWH	2	50
52	UFH	2	50
53	Rivaroxaban	1	50
53	LMWH	0	25
54	Fondaparinux	44	784
54	LMWH	65	796
55	Rivaroxaban	14	60
55	LMWH	31	70
56	Rivaroxaban	61	965
56	LMWH	86	959
57	Betrixaban	9	65
57	LMWH	4	40
58	Rivaroxaban	8	96
58	LMWH	17	99
59	Rivaroxaban	1	89
59	LMWH	4	89
60	Rivaroxaban	1	60
60	LMWH	3	60
61	LMWH	21	76
61	Apixaban	12	83
62	Rivaroxaban	3	15
62	LMWH	4	15
63	Rivaroxaban	4	75
63	LMWH	3	70
64	Fondaparinux	6	84
64	LMWH	5	83
65	LMWH	6	45
65	Rivaroxaban	7	45
66	LMWH	6	43
66	Rivaroxaban	1	43
67	Rivaroxaban	3	102
67	LMWH	14	112
67	Aspirin	18	110

DVT = deep vein thrombosis, LMWH = low molecular weight heparin, UFH = unfractionated heparin.

**Table 5:** The *P* score of anticoagulants’ efficacy for prevention of DVT.

Anticoagulants	*P* score	Rank
Apixaban	.940	1
Edoxaban	.917	2
Fondaparinux	.750	3
Rivaroxaban	.731	4
Darexaban	.621	5
Dabigatran	.392	6
LMWH	.358	7
Aspirin	.310	8
UFH	.233	9
Betrixaban	.216	10
Warfarin	.033	11

DVT = deep vein thrombosis, LMWH = low molecular weight heparin, UFH = unfractionated heparin.

**Table 6:** Characteristics of anticoagulants’ efficacy for prevention of PE.

Study	Treatment	Responder	Sample size
1	LMWH	3	398
1	Aspirin	0	380
2	Rivaroxaban	4	902
2	Aspirin	3	902
3	Rivaroxaban	4	815
3	Aspirin	4	805
4	Rivaroxaban	0	42
4	LMWH	0	42
5	Rivaroxaban	0	114
5	LMWH	0	114
6	Fondaparinux	1	517
6	LMWH	4	517
7	Rivaroxaban	0	60
7	LMWH	0	60
8	Dabigatran	0	297
8	LMWH	0	300
9	Dabigatran	0	675
9	LMWH	1	685
10	Dabigatran	5	1137
10	LMWH	3	1142
11	Rivaroxaban	0	59
11	LMWH	0	107
12	Rivaroxaban	4	1595
12	LMWH	1	1558
13	Darexaban	0	120
13	LMWH	0	127
14	Dabigatran	1	1001
14	LMWH	2	992
15	Dabigatran	1	1001
15	LMWH	2	992
16	Warfarin	0	122
16	LMWH	0	108
17	Edoxaban	0	299
17	LMWH	0	295
18	Edoxaban	0	255
18	LMWH	0	248
19	Dabigatran	6	604
19	LMWH	5	643
20	LMWH	1	813
20	UFH	1	815
21	Fondaparinux	1	277
21	LMWH	5	297
22	Rivaroxaban	1	864
22	LMWH	4	869
23	LMWH	1	125
23	UFH	2	134
24	Rivaroxaban	2	350
24	LMWH	1	351
25	Fondaparinux	2	1129
25	LMWH	2	1123
26	Apixaban	0	105
26	LMWH	2	109
26	Warfarin	0	109
27	Rivaroxaban	0	824
27	LMWH	4	878
28	Fondaparinux	1	360
28	LMWH	0	223
28	UFH	0	72
29	Fondaparinux	0	261
29	LMWH	0	148
29	UFH	0	32
30	LMWH	0	45
30	Dabigatran	0	45
31	Rivaroxaban	1	50
31	LMWH	2	50
32	Rivaroxaban	0	48
32	LMWH	0	36
33	Rivaroxaban	0	80
33	LMWH	0	80
34	LMWH	0	50
34	UFH	0	50
35	Fondaparinux	5	1126
35	LMWH	1	1128
36	Rivaroxaban	0	60
36	LMWH	0	70
37	Rivaroxaban	5	1526
37	LMWH	8	1508
38	Betrixaban	1	65
38	LMWH	0	40
39	LMWH	0	76
39	Apixaban	0	83
40	Rivaroxaban	0	15
40	LMWH	0	15
41	Fondaparinux	0	84
41	LMWH	0	83
42	Rivaroxaban	0	102
42	LMWH	0	112
42	Aspirin	0	110

LMWH = low molecular weight heparin, PE = pulmonary embolism, UFH = unfractionated heparin.

**Table 7:** The *P* score of anticoagulants’ efficacy for prevention of PE.

Anticoagulants	*P* score	Rank
Warfarin	.716	1
Apixaban	.708	2
Aspirin	.671	3
Rivaroxaban	.580	4
Fondaparinux	.545	5
Edoxaban	.461	6
Darexaban	.455	7
LMWH	.400	8
Dabigatran	.395	9
Betrixaban	.325	10
UFH	.244	11

LMWH = low molecular weight heparin, PE = pulmonary embolism, UFH = unfractionated heparin.

### 5.3. Grading the evidence of the network meta-analysis using CINeMA

After the assessment of 6 domains by CINeMA (Table S1, Supplemental Digital Content, http://links.lww.com/MD/I302; Table S2, Supplemental Digital Content, http://links.lww.com/MD/I303; Table S3, Supplemental Digital Content, http://links.lww.com/MD/I304; Table S4, Supplemental Digital Content, http://links.lww.com/MD/I305; Table S5, Supplemental Digital Content, http://links.lww.com/MD/I306; and Table S6, Supplemental Digital Content, http://links.lww.com/MD/I307), every included study’s confidence rating was assigned as follows: high = 0; moderate = −1; low = −2; very low ≤ −3. The entire confidence in evidence was obtained by the weighted average algorithm of confidence rating of every included study. The entire confidence in the evidence of anticoagulants for the prevention of VTE was low.

## 6. Discussion

VTE is a frequent and increasing disease, which is associated with severe venous disease and high treatment costs.^[70]^ The main reason for the occurrence of VTE is that after joint replacement, the human body is in a hypercoagulable state, where the coagulation mechanism is activated, procoagulant substances such as thromboxane and fibrinogen increase, and inflammation and edema of the surgical site tissue compress the blood vessels, resulting in slow local blood flow.^[71]^ Therefore, to prevent the occurrence of VTE after THA or TKA, it is not enough to only rely on bed rest, use of pneumatic compression, wearing compression stockings, and other general measures; systematic anticoagulant therapy must be given.

In this study, we reviewed the efficacy of 11 anticoagulants in patients undergoing THA or TKA. Evidence was compiled from direct and indirect comparisons to evaluate the efficacy. For the prevention of DVT, the results of the league table and *P* score showed that apixaban, edoxaban, fondaparinux, rivaroxaban, and darexaban were the most effective anticoagulants for patients undergoing THA or TKA. Due to the relatively low incidence of PE, the results of the league table and *P* score showed that there was no difference in the efficacy among the anticoagulants for the prevention of PE. All these effective anticoagulants belong to new oral anticoagulants (NOACs) (apixaban, edoxaban, rivaroxaban, and darexaban). NOACs represent novel direct-acting medications that directly inhibit factor Xa or factor IIa.^[72]^ These drugs have been approved for the prevention of VTE in patients after elective hip or knee arthroplasty in the European Union and many other countries worldwide.^[73]^ Compared with other traditional anticoagulants, NOACs have various advantages, such as the absence of food interactions, few strong drug interactions, predictable pharmacokinetics and pharmacodynamics, rapid onset and offset of action, and absence of the need for laboratory monitoring.^[74,75]^ However, NOACs have disadvantages, such as contraindication or dose reduction in patients with chronic kidney disease or hepatic disease, absence of a specific test, the potential for overuse, lack of a specific antidote in case of major bleeding, and high costs.^[74,75]^

At the same time, the short half-lives of NOACs can be considered both an advantage and a disadvantage under various circumstances. For example, the advantage of the short half-life of an NOAC may be relevant for emergency surgery and in cases of bleeding due to accumulation of the drug in the blood, whereas the short half-life is a disadvantage if the patient forgets to take the drug, which could put the patient at risk^[74]^; therefore, many traditional anticoagulants can be replaced. This study showed that fondaparinux had a high efficacy for the prevention of DVT. Fondaparinux, a synthetic pentasaccharide, is the first drug in a new class of antithrombotic agents – selective factor Xa inhibitors.^[76]^ Fondaparinux is completely absorbed following subcutaneous injection, and its activity is higher than that of LMWH (about 700 units/mg and 100 units/mg, respectively).^[77]^ The half-life of fondaparinux is 17 hours, so it is suitable for daily dosing.^[78]^ Fondaparinux undergoes renal clearance and therefore requires dose reduction or replacement in patients with poor renal function.^[77]^

This article had some limitations. First, studies in languages other than English and Chinese were excluded, which may have affected the comprehensiveness of the included studies. Second, the entire confidence in the collected evidence as assessed by CINeMA was low, so the results should be interpreted with caution.

## 7. Conclusion

In this study, we provided a useful reference for the selection of anticoagulants for the prevention of VTE after THA or TKA. Apixaban, edoxaban, fondaparinux, rivaroxaban, and darexaban showed the best efficacy. However, more high-quality studies are needed to confirm the above conclusions.

## Author contributions

**Conceptualization:** Zhihao Huang, Xinru Xu, Dan Xu.

**Formal analysis:** Zhihao Huang, Xinru Xu, Miao Zou.

**Investigation:** Xinru Xu.

**Methodology:** Zhihao Huang, Pengfei Zhao.

**Project administration:** Zhihao Huang.

**Software:** Zhihao Huang.

**Supervision:** Zhihao Huang, Xinru Xu.

**Visualization:** Xinru Xu, Dan Xu.

**Writing – original draft:** Zhihao Huang, Xinru Xu.

**Writing – review and editing:** Zhihao Huang, Xinru Xu, Dan Xu, Pengfei Zhao, Miao Zou.

## Supplementary Material


