# Supplementary material for: Efficacy of 11 anticoagulants for the prevention of venous thromboembolism after total hip or knee arthroplasty: A systematic review and network meta-analysis
Source: Medicine (Baltimore). 2023 Jan 13;102(2):e32635. doi: 10.1097/MD.0000000000032635 (PMC9839234; doi:10.1097/MD.0000000000032635)
Supplement: Supplementary file 1 [file medi-102-e32635-s001.pdf]

Supplemental Method 1 Electronic search strategies.

PubMed

#1 ((((Joint replacement[Title/Abstract]) OR (Knee replacement[Title/Abstract])) OR (Hip replacement[Title/Abstract])) OR (Joint prosthesis implantation[Title/Abstract])) OR (Arthroplasty[Title/Abstract])

#2 (((((Venous thromboembolism[Title/Abstract]) OR (VTE[Title/Abstract])) OR (Deep venous thrombosis[Title/Abstract])) OR (DVT[Title/Abstract])) OR (Pulmonary embolism[Title/Abstract])) OR (PE[Title/Abstract])

#3 #1 AND #2

#4 (((Aspirin[Title/Abstract]) OR (Acetylsalicylic acid[Title/Abstract])) OR (2-(Acetyloxy)benzoic acid[Title/Abstract])) OR (Acylpyrin[Title/Abstract])) OR (Aloxiprimum[Title/Abstract])) OR (Colfarit[Title/Abstract])) OR (Dispril[Title/Abstract])) OR (Easprin[Title/Abstract])) OR (Ecotrin[Title/Abstract])) OR (Endosprin[Title/Abstract])) OR (Magnecyl[Title/Abstract])) OR (Micristin[Title/Abstract])) OR (Polopirin[Title/Abstract])) OR (Polopiryna[Title/Abstract])) OR (Solprin[Title/Abstract])) OR (Solupsan[Title/Abstract])) OR (Zorprin[Title/Abstract])) OR (Acetysal[Title/Abstract])) OR (Warfarin[Title/Abstract])) OR (4-Hydroxy-3-(3-oxo-1-phenylbutyl)-2H-1-benzopyran-2-one[Title/Abstract])) OR (Aldocumar[Title/Abstract])) OR (Warfant[Title/Abstract])) OR (Coumadin[Title/Abstract])) OR (Marevan[Title/Abstract])) OR (Coumadine[Title/Abstract])) OR (Tedicumar[Title/Abstract])) OR (Tranexamic acid[Title/Abstract])) OR (AMCHA[Title/Abstract])) OR (trans-4-(Aminomethyl)cyclohexanecarboxylic acid[Title/Abstract])) OR (AMCA[Title/Abstract])) OR (Anvitoff[Title/Abstract])) OR (Cyklokapron[Title/Abstract])) OR (Ugurol[Title/Abstract])) OR (KABI 2161[Title/Abstract])) OR (Spotof[Title/Abstract])) OR (Transamin[Title/Abstract])) OR (Amchafibrin[Title/Abstract])) OR (Exacyl[Title/Abstract])) OR (Fondaparinux[Title/Abstract])) OR (Quixidar[Title/Abstract])) OR (Arixtra[Title/Abstract])) OR (Ximelagatran[Title/Abstract])) OR (Melagatran[Title/Abstract])) OR (Exanta[Title/Abstract])) OR (H 376 95[Title/Abstract])) OR (Dextran[Title/Abstract])) OR (Hemodex[Title/Abstract])) OR (Hyskon[Title/Abstract])) OR (Infukoll[Title/Abstract])) OR (Macrodex[Title/Abstract])) OR (Polyglucin[Title/Abstract])) OR (Promit[Title/Abstract])) OR (Rheodextran[Title/Abstract])) OR (Rheoisodex[Title/Abstract])) OR (Rheomacrodex[Title/Abstract])) OR (Rheopolyglucin[Title/Abstract])) OR (Rondex[Title/Abstract])) OR (Saviosol[Title/Abstract])) OR (Apixaban[Title/Abstract])) OR (Eliquis[Title/Abstract])) OR (BMS 562247[Title/Abstract])) OR (Rivaroxaban[Title/Abstract])) OR (Xarelto[Title/Abstract])) OR (BAY 597939[Title/Abstract])) OR (Edoxaban[Title/Abstract])) OR (Savaysa[Title/Abstract])) OR (DU-176[Title/Abstract])) OR (Betrixaban[Title/Abstract])) OR (Bevyxxa[Title/Abstract])) OR (PRT054021[Title/Abstract])) OR (Letaxaban[Title/Abstract])) OR (TAK-442[Title/Abstract])) OR (Darexaban[Title/Abstract])) OR (YM150[Title/Abstract])) OR (Dabigatran[Title/Abstract])) OR (BIBR 1048[Title/Abstract])) OR (Pradaxa[Title/Abstract])) OR (Heparin[Title/Abstract])) OR (Heparinic acid[Title/Abstract])) OR (Liquaemin[Title/Abstract])) OR (LMWH[Title/Abstract])) OR (Enoxaparin[Title/Abstract])) OR (Dalteparin[Title/Abstract])) OR (Heparinoid[Title/Abstract])) OR (Atheroid[Title/Abstract])) OR (Ateroid[Title/Abstract])

#5 randomized controlled trial[Publication Type] OR randomized[Title/Abstract] OR placebo[Title/Abstract]

#6 #3 AND #5

Cochran Library

#1 (Joint replacement):ab,ti,kw OR (Knee replacement):ab,ti,kw OR (Hip replacement):ab,ti,kw OR (Joint prosthesis implantation):ab,ti,kw OR (Arthroplasty):ab,ti,kw

#2 (Venous thromboembolism):ab,ti,kw OR (VTE):ab,ti,kw OR (Deep venous thrombosis):ab,ti,kw OR (DVT):ab,ti,kw OR (Pulmonary embolism):ab,ti,kw OR (PE):ab,ti,kw

#3 #1 AND #2

#4 (Aspirin):ab,ti,kw OR (Acetylsalicylic acid):ab,ti,kw OR (Acylpyrin):ab,ti,kw OR (Aloxiprimum):ab,ti,kw OR (Colfarit):ab,ti,kw OR (Dispril):ab,ti,kw OR (Easprin):ab,ti,kw OR (Ecotrin):ab,ti,kw OR (Endosprin):ab,ti,kw OR (Magnecyl):ab,ti,kw OR (Micristin):ab,ti,kw OR (Polopirin):ab,ti,kw OR (Polopiryna):ab,ti,kw OR (Solprin):ab,ti,kw OR (Solupsan):ab,ti,kw OR (Zorprin):ab,ti,kw OR (Acetysal):ab,ti,kw OR (Warfarin):ab,ti,kw OR (Aldocumar):ab,ti,kw OR (Warfant):ab,ti,kw OR (Coumadin):ab,ti,kw OR (Marevan):ab,ti,kw OR (Coumadine):ab,ti,kw OR (Tedicumar):ab,ti,kw OR (Tranexamic acid):ab,ti,kw OR (AMCHA):ab,ti,kw OR (AMCA):ab,ti,kw OR (Anvitoff):ab,ti,kw OR (Cyklokapron):ab,ti,kw OR (Ugurol):ab,ti,kw OR (KABI 2161):ab,ti,kw OR (Spotof):ab,ti,kw OR (Transamin):ab,ti,kw OR (Amchafibrin):ab,ti,kw OR (Exacyl):ab,ti,kw OR (Fondaparinux):ab,ti,kw OR (Quixidar):ab,ti,kw OR (Arixtra):ab,ti,kw OR (Ximelagatran):ab,ti,kw OR (Melagatran):ab,ti,kw OR (Exanta):ab,ti,kw OR (H 376 95):ab,ti,kw OR (Dextran):ab,ti,kw OR (Hemodex):ab,ti,kw OR (Hyskon):ab,ti,kw OR (Infukoll):ab,ti,kw OR (Macrodex):ab,ti,kw OR (Polyglucin):ab,ti,kw OR (Promit):ab,ti,kw OR (Rheodextran):ab,ti,kw OR (Rheoisodex):ab,ti,kw OR (Rheomacrodex):ab,ti,kw OR (Rheopolyglucin):ab,ti,kw OR (Rondex):ab,ti,kw OR (Saviosol):ab,ti,kw OR (Apixaban):ab,ti,kw OR (Eliquis):ab,ti,kw OR (BMS 562247):ab,ti,kw OR (Rivaroxaban):ab,ti,kw OR (Xarelto):ab,ti,kw OR (BAY 597939):ab,ti,kw OR (Edoxaban):ab,ti,kw OR (Savaysa):ab,ti,kw OR (DU-176):ab,ti,kw OR (Betrixaban):ab,ti,kw OR (Bevyxxa):ab,ti,kw OR (PRT054021):ab,ti,kw OR (Letaxaban):ab,ti,kw OR (TAK-442):ab,ti,kw OR (Darexaban):ab,ti,kw OR (YM150):ab,ti,kw OR (Dabigatran):ab,ti,kw OR (BIBR 1048):ab,ti,kw OR (Pradaxa):ab,ti,kw OR (Heparin):ab,ti,kw OR (Heparinic acid):ab,ti,kw OR (Liquaemin):ab,ti,kw OR (LMWH):ab,ti,kw OR (Enoxaparin):ab,ti,kw OR (Dalteparin):ab,ti,kw OR (Heparinoid):ab,ti,kw OR (Atheroid):ab,ti,kw OR (Ateroid):ab,ti,kw

#5 (randomized controlled trial):ab,ti,kw OR (randomized):ab,ti,kw OR (placebo):ab,ti,kw

#6 #3 AND #4 AND #5

Embase

#1 'Joint replacement':ab,ti OR 'Knee replacement':ab,ti OR 'Hip replacement':ab,ti OR 'Joint prosthesis implantation':ab,ti OR 'Arthroplasty':ab,ti

#2 'Venous thromboembolism':ab,ti OR 'VTE':ab,ti OR 'Deep venous thrombosis':ab,ti OR 'DVT':ab,ti OR 'Pulmonary embolism':ab,ti OR 'PE':ab,ti

#3 #1 AND #2

#4 'Aspirin':ab,ti OR 'Acetylsalicylic acid':ab,ti OR '2-(Acetyloxy)benzoic acid':ab,ti OR 'Acylpyrin':ab,ti OR 'Aloxiprimum':ab,ti OR 'Colfarit':ab,ti OR 'Dispril':ab,ti OR 'Easprin':ab,ti OR 'Ecotrin':ab,ti OR 'Endosprin':ab,ti OR 'Magnecyl':ab,ti OR 'Micristin':ab,ti OR 'Polopirin':ab,ti OR 'Polopiryna':ab,ti OR 'Solprin':ab,ti OR 'Solupsan':ab,ti OR 'Zorprin':ab,ti OR 'Acetysal':ab,ti OR 'Warfarin':ab,ti OR '4-Hydroxy-3-(3-oxo-1-phenylbutyl)-2H-1-benzopyran-2-o

ne':ab,ti OR 'Aldocumar':ab,ti OR 'Warfant':ab,ti OR 'Coumadin':ab,ti OR 'Marevan':ab,ti OR 'Coumadine':ab,ti OR 'Tedicumar':ab,ti OR 'Tranexamic acid':ab,ti OR 'AMCHA':ab,ti OR 'trans-4-(Aminomethyl)cyclohexanecarboxylic acid':ab,ti OR 'AMCA':ab,ti OR 'Anvitoff':ab,ti OR 'Cyklokapron':ab,ti OR 'Ugurol':ab,ti OR 'KABI 2161':ab,ti OR 'Spotof':ab,ti OR 'Transamin':ab,ti OR 'Amchafibrin':ab,ti OR 'Exacyl':ab,ti OR 'Fondaparinux':ab,ti OR 'Quixidar':ab,ti OR 'Arixtra':ab,ti OR 'Ximelagatran':ab,ti OR 'Melagatran':ab,ti OR 'Exanta':ab,ti OR 'H 376 95':ab,ti OR 'Dextran':ab,ti OR 'Hemodex':ab,ti OR 'Hyskon':ab,ti OR 'Infukoll':ab,ti OR 'Macrodex':ab,ti OR 'Polyglucin':ab,ti OR 'Promit':ab,ti OR 'Rheodextran':ab,ti OR 'Rheoisodex':ab,ti OR 'Rheomacrodex':ab,ti OR 'Rheopolyglucin':ab,ti OR 'Rondex':ab,ti OR 'Saviosol':ab,ti OR 'Apixaban':ab,ti OR 'Eliquis':ab,ti OR 'BMS 562247':ab,ti OR 'Rivaroxaban':ab,ti OR 'Xarelto':ab,ti OR 'BAY 597939':ab,ti OR 'Edoxaban':ab,ti OR 'Savaysa':ab,ti OR 'DU-176':ab,ti OR 'Betrixaban':ab,ti OR 'Bevyxxa':ab,ti OR 'PRT054021':ab,ti OR 'Letaxaban':ab,ti OR 'TAK-442':ab,ti OR 'Darexaban':ab,ti OR 'YM150':ab,ti OR 'Dabigatran':ab,ti OR 'BIBR 1048':ab,ti OR 'Pradaxa':ab,ti OR 'Heparin':ab,ti OR 'Heparinic acid':ab,ti OR 'Liquaemin':ab,ti OR 'LMWH':ab,ti OR 'Enoxaparin':ab,ti OR 'Dalteparin':ab,ti OR 'Heparinoid':ab,ti OR 'Atheroid':ab,ti OR 'Ateroid':ab,ti
#5 'randomized controlled trial':ab,ti OR 'randomized':ab,ti OR 'placebo':ab,ti
#6 #3 AND #4 AND #5

Web of Science

#1 TS=(Joint replacement or Knee replacement or Hip replacement or Joint prosthesis implantation or Arthroplasty)
#2 TS=(Venous thromboembolism or VTE or Deep venous thrombosis or DVT or Pulmonary embolism or PE)
#3 #1 AND #2
#4 TS=(Aspirin or Acetylsalicylic acid or 2-(Acetyloxy)benzoic acid or Acylpyrin or Aloxiprimum or Colfarit or Dispril or Easprin or Ecotrin or Endosprin or Magnecyl or Micristin or Polopirin or Polopiryna or Solprin or Solupsan or Zorprin or Acetysal or Warfarin or 4-Hydroxy-3-(3-oxo-1-phenylbutyl)-2H-1-benzopyran-2-one or Aldocumar or Warfant or Coumadin or Marevan or Coumadine or Tedicumar or Tranexamic acid or AMCHA or trans-4-(Aminomethyl)cyclohexanecarboxylic acid or AMCA or Anvitoff or Cyklokapron or Ugurol or KABI 2161 or Spotof or Transamin or Amchafibrin or Exacyl or Fondaparinux or Quixidar or Arixtra or Ximelagatran or Melagatran or Exanta or H 376 95 or Dextran or Hemodex or Hyskon or Infukoll or Macrodex or Polyglucin or Promit or Rheodextran or Rheoisodex or Rheomacrodex or Rheopolyglucin or Rondex or Saviosol or Apixaban or Eliquis or BMS 562247 or Rivaroxaban or Xarelto or BAY 597939 or Edoxaban or Savaysa or DU-176 or Betrixaban or Bevyxxa or PRT054021 or Letaxaban or TAK-442 or Darexaban or YM150 or Dabigatran or BIBR 1048 or Pradaxa or Heparin or Heparinic acid or Liquaemin or LMWH or Enoxaparin or Dalteparin or Heparinoid or Atheroid or Ateroid)
#5 TS=(randomized controlled trial or randomized or placebo)
#6 #3 AND #4 AND #5

China National Knowledge Infrastructure

#1 关节置换 + 膝关节置换 + 髋关节置换
#2 静脉血栓 + 深静脉血栓 + 肺血栓
#3 #1 AND #2

#4 阿司匹林 + 华法林 + 氨甲环酸 + 磺达肝癸钠 + 希美拉加群 + 美拉加群 + 右旋糖酐 + 阿哌沙班 + 利伐沙班 + 依度沙班 + 贝曲沙班 + 来他沙班 + YM150 + 达比加群酯 + 肝素 + 类肝素
#5 随机对照 + 随机 + RCT
#6 #3 AND #4 AND #5

Wanfang Data

主题:(关节置换 or 膝关节置换 or 髋关节置换) and 主题:(静脉血栓 or 深静脉血栓 or 肺血栓) and 主题:(阿司匹林 or 华法林 or 氨甲环酸 or 磺达肝癸钠 or 希美拉加群 or 美拉加群 or 右旋糖酐 or 阿哌沙班 or 利伐沙班 or 依度沙班 or 贝曲沙班 or 来他沙班 or YM150 or 达比加群酯 or 肝素 or 类肝素) and 主题:(随机对照 or 随机 or RCT)

VIP

#1 关节置换 + 膝关节置换 + 髋关节置换
#2 静脉血栓 + 深静脉血栓 + 肺血栓
#3 阿司匹林 + 华法林 + 氨甲环酸 + 磺达肝癸钠 + 希美拉加群 + 美拉加群 + 右旋糖酐 + 阿哌沙班 + 利伐沙班 + 依度沙班 + 贝曲沙班 + 来他沙班 + YM150 + 达比加群酯 + 肝素 + 类肝素
#4 随机对照 + 随机 + RCT
#5 #1 与 #2 与 #3 与 #4

China Biology Medicine

#1 "关节置换"[常用字段:智能] OR "膝关节置换"[常用字段:智能] OR "髋关节置换"[常用字段:智能]
#2 "静脉血栓"[常用字段:智能] OR "深静脉血栓"[常用字段:智能] OR "肺血栓"[常用字段:智能]
#3 #1 AND #2
#4 "阿司匹林"[常用字段:智能] OR "华法林"[常用字段:智能] OR "氨甲环酸"[常用字段:智能] OR "磺达肝癸钠"[常用字段:智能] OR "希美拉加群"[常用字段:智能] OR "美拉加群"[常用字段:智能] OR "右旋糖酐"[常用字段:智能] OR "阿哌沙班"[常用字段:智能] OR "利伐沙班"[常用字段:智能] OR "依度沙班 "[常用字段:智能] OR "贝曲沙班"[常用字段:智能] OR "来他沙班"[常用字段:智能] OR "YM150"[常用字段:智能] OR "达比加群酯"[常用字段:智能] OR "肝素"[常用字段:智能] OR "类肝素"[常用字段:智能]
#5 "随机对照"[常用字段:智能] OR "随机"[常用字段:智能] OR "RCT"[常用字段:智能]
#6 #3 AND #4 AND #5
