# Supplementary material for: Efficacy of 11 anticoagulants for the prevention of venous thromboembolism after total hip or knee arthroplasty: A systematic review and network meta-analysis
Source: Medicine (Baltimore). 2023 Jan 13;102(2):e32635. doi: 10.1097/MD.0000000000032635 (PMC9839234; doi:10.1097/MD.0000000000032635)
Supplement: Supplementary file 2 [file medi-102-e32635-s002.pdf]

Supplemental Table 1 Jadad quality score of the included studies on anticoagulants' efficacy for prevention of DVT.

NO.	Study	random sequence production	allocation concealment	blinding method	withdrawals and dropouts	total score
1	Anderson 2013	2	1	2	1	6
2	Anderson 2018	2	1	2	1	6
3	Anderson 2018	2	1	2	1	6
4	Argun 2013	1	1	0	1	3
5	Bai 2018	2	1	0	1	4
6	Bai 2020	2	1	0	1	4
7	Bai 2021	2	1	0	1	4
8	Bauer 2001	2	1	2	1	6
9	Bonneux 2006	2	1	0	1	4
10	Colleoni 2008	2	1	0	1	4
11	Ding 2014	2	1	0	1	4
12	Eriksson 2005	2	1	2	1	6
13	Eriksson 2006	1	2	2	1	6
14	Eriksson 2007	2	1	2	1	6
15	Eriksson 2007	2	1	2	1	6
16	Eriksson 2007	2	2	2	1	7
17	Eriksson 2007	2	2	2	1	7
18	Eriksson 2007	1	2	0	1	4
19	Eriksson 2007	2	1	0	1	4
20	Eriksson 2008	2	1	2	1	6
21	Eriksson 2010	2	1	1	1	5
22	Eriksson 2011	2	2	2	1	7
23	Eriksson 2011	2	2	2	1	7
24	Fizgerald 2001	2	1	2	1	6
25	Fuji 2014	2	1	2	1	6
26	Fuji 2014	1	1	0	1	3
27	Fuji 2014	1	1	0	1	3
28	Fuji 2015	1	1	2	1	5
29	Gao 2011	0	0	0	1	1
30	Gao 2016	2	1	0	1	4
31	Ginsberg 2009	2	2	2	1	7
32	Guo 2018	1	1	0	1	3
33	Hass 2006	2	1	2	1	6
34	Hosaka 2013	0	0	0	1	1
35	Hull 2000	2	1	0	1	4
36	Jiang 2019	1	1	0	1	3
37	Kakkar 2000	1	1	2	1	5
38	Kakkar 2008	2	2	2	1	7
39	Kim 2016	2	1	2	1	6
40	Lassen 2002	2	0	2	1	5
41	Lassen 2007	2	1	2	1	6
42	Lassen 2008	2	1	1	1	5
43	Li 2018	1	1	0	1	3
44	Migita 2014	0	0	0	1	1
45	Migita 2014	0	0	0	1	1
46	Mirdamadi 2014	2	1	1	1	5
47	Qin 2016	2	1	0	1	4
48	Quan 2010	2	1	0	1	4
49	Rahman 2020	2	1	0	1	4
50	Raskob 2010	1	1	2	1	5
51	Ren 2021	1	1	0	1	3
52	Senaran 2005	1	1	0	1	3
53	Shi 2014	1	1	0	1	3
54	Turpie 2002	2	1	2	1	6
55	Turpie 2005	2	2	2	1	7
56	Turpie 2009	2	2	2	1	7
57	Turpie 2009	2	2	0	1	5
58	Wang 2014	0	0	1	1	2
59	Wang 2017	2	1	0	1	4
60	Wang 2020	2	1	1	1	5
61	Weitz 2020	2	0	0	1	3
62	Wu 2013	2	1	0	1	4
63	Yang 2013	1	1	0	1	3
64	Yokote 2011	1	2	1	1	5
65	Zhang 2017	0	0	0	1	1
66	Zhang 2020	1	1	0	1	3
67	Zou 2014	2	1	0	1	4
