# Supplementary material for: Efficacy of 11 anticoagulants for the prevention of venous thromboembolism after total hip or knee arthroplasty: A systematic review and network meta-analysis
Source: Medicine (Baltimore). 2023 Jan 13;102(2):e32635. doi: 10.1097/MD.0000000000032635 (PMC9839234; doi:10.1097/MD.0000000000032635)
Supplement: Supplementary file 3 [file medi-102-e32635-s003.pdf]

Supplemental Table 2 Jadad quality score of the included studies on anticoagulants' efficacy for prevention of PE.

NO.	Study ID	random sequence production	allocation concealment	blinding method	withdrawals and dropouts	total score
1	Anderson 2013	2	1	2	1	6
2	Anderson 2018	2	1	2	1	6
3	Anderson 2018	2	1	2	1	6
4	Bai 2020	2	1	0	1	4
5	Bai 2021	2	1	0	1	4
6	Bauer 2001	2	1	2	1	6
7	Ding 2014	2	1	0	1	4
8	Eriksson 2005	2	1	2	1	6
9	Eriksson 2007	2	1	2	1	6
10	Eriksson 2007	2	2	2	1	7
11	Eriksson 2007	1	2	0	1	4
12	Eriksson 2008	2	1	2	1	6
13	Eriksson 2010	2	1	1	1	5
14	Eriksson 2011	2	2	2	1	7
15	Eriksson 2011	2	2	2	1	7
16	Fizgerald 2001	2	1	2	1	6
17	Fuji 2014	2	1	2	1	6
18	Fuji 2015	1	1	2	1	5
19	Ginsberg 2009	2	2	2	1	7
20	Hass 2006	2	1	2	1	6
21	Hosaka 2013	0	0	0	1	1
22	Kakkar 2000	1	1	2	1	5
23	Kakkar 2008	2	2	2	1	7
24	Kim 2016	2	1	2	1	6
25	Lassen 2002	2	0	2	1	5
26	Lassen 2007	2	1	2	1	6
27	Lassen 2008	2	1	1	1	5
28	Migita 2014	0	0	0	1	1
29	Migita 2014	0	0	0	1	1
30	Mirdamadi 2014	2	1	1	1	5
31	Qin 2016	2	1	0	1	4
32	Quan 2010	2	1	0	1	4
33	Rahman 2020	2	1	0	1	4
34	Senaran 2005	1	1	0	1	3
35	Turpie 2002	2	1	2	1	6
36	Turpie 2005	2	2	2	1	7
37	Turpie 2009	2	2	2	1	7
38	Turpie 2009	2	2	0	1	5
39	Weitz 2020	2	0	0	1	3
40	Wu 2013	2	1	0	1	4
41	Yokote 2011	1	2	1	1	5
42	Zou 2014	2	1	0	1	4
