# Supplementary material for: Efficacy of 11 anticoagulants for the prevention of venous thromboembolism after total hip or knee arthroplasty: A systematic review and network meta-analysis
Source: Medicine (Baltimore). 2023 Jan 13;102(2):e32635. doi: 10.1097/MD.0000000000032635 (PMC9839234; doi:10.1097/MD.0000000000032635)
Supplement: Supplementary file 4 [file medi-102-e32635-s004.pdf]

Supplemental Table 3 DVT outcome’s data format that be used as input to CINeMA. Rob and indirectness can take either 1, 2, and 3 values for low, moderate, and high risk of bias or level of indirectness.

NO.	study	r	n	rob	indirectness
1	LMWH	2	398	1	1
1	aspirin	1	380	1	1
2	rivaroxaban	3	902	1	1
2	aspirin	2	902	1	1
3	rivaroxaban	3	815	1	1
3	aspirin	4	805	1	1
4	fondaparinux	0	55	3	1
4	LMWH	1	53	3	1
5	rivaroxaban	4	98	2	1
5	LMWH	11	98	2	1
6	rivaroxaban	14	42	2	1
6	LMWH	10	42	2	1
7	rivaroxaban	2	114	2	1
7	LMWH	4	114	2	1
8	fondaparinux	45	361	1	1
8	LMWH	98	361	1	1
9	fondaparinux	2	55	2	1
9	LMWH	1	54	2	1
10	aspirin	1	14	2	1
10	rivaroxaban	2	18	2	1
11	rivaroxaban	5	60	2	1
11	LMWH	6	60	2	1
12	dabigatran	39	297	1	1
12	LMWH	72	300	1	1
13	rivaroxaban	2	29	1	1
13	LMWH	18	106	1	1
14	dabigatran	181	503	1	1
14	LMWH	184	511	1	1
15	dabigatran	1	675	1	1
15	LMWH	8	685	1	1
16	dabigatran	40	874	1	1
16	LMWH	56	894	1	1
17	dabigatran	6	1137	1	1
17	LMWH	1	1142	1	1
18	rivaroxaban	6	59	2	1
18	LMWH	18	107	2	1
19	darexaban	5	27	2	1
19	LMWH	12	31	2	1
20	rivaroxaban	12	1595	1	1

20	LMWH	53	1558	1	1
21	darexaban	16	120	1	1
21	LMWH	24	127	1	1
22	dabigatran	60	791	1	1
22	LMWH	67	783	1	1
23	dabigatran	0	1001	1	1
23	LMWH	4	992	1	1
24	warfarin	72	122	1	1
24	LMWH	41	108	1	1
25	edoxaban	22	299	1	1
25	LMWH	41	295	1	1
26	darexaban	4	136	3	1
26	LMWH	2	82	3	1
27	darexaban	11	71	3	1
27	LMWH	14	66	3	1
28	edoxaban	6	255	1	1
28	LMWH	17	248	1	1
29	LMWH	37	166	3	1
29	aspirin	28	120	3	1
30	LMWH	5	54	2	1
30	rivaroxaban	4	54	2	1
31	dabigatran	188	604	1	1
31	LMWH	163	643	1	1
32	LMWH	3	30	3	1
32	rivaroxaban	1	30	3	1
33	LMWH	200	813	1	1
33	UFH	204	815	1	1
34	fondaparinux	13	275	3	1
34	LMWH	18	296	3	1
35	LMWH	80	673	2	1
35	warfarin	81	338	2	1
36	apixaban	6	110	3	1
36	LMWH	22	110	3	1
37	rivaroxaban	14	864	1	1
37	LMWH	71	869	1	1
38	LMWH	9	101	1	1
38	UFH	24	116	1	1
39	rivaroxaban	24	350	1	1
39	LMWH	23	351	1	1
40	fondaparinux	36	908	1	1
40	LMWH	83	918	1	1
41	apixaban	5	105	1	1
41	LMWH	15	109	1	1

41	warfarin	29	109	1	1
42	rivaroxaban	79	824	1	1
42	LMWH	160	878	1	1
43	rivaroxaban	0	50	3	1
43	LMWH	4	50	3	1
44	fondaparinux	60	360	3	1
44	LMWH	59	223	3	1
44	UFH	24	72	3	1
45	fondaparinux	17	261	3	1
45	LMWH	17	148	3	1
45	UFH	5	32	3	1
46	LMWH	1	45	1	1
46	dabigatran	1	45	1	1
47	rivaroxaban	3	50	2	1
47	LMWH	5	50	2	1
48	rivaroxaban	10	48	2	1
48	LMWH	9	36	2	1
49	rivaroxaban	8	80	2	1
49	LMWH	0	80	2	1
50	LMWH	20	144	1	1
50	edoxaban	2	158	1	1
51	aspirin	3	34	3	1
51	rivaroxaban	3	36	3	1
52	LMWH	2	50	3	1
52	UFH	2	50	3	1
53	rivaroxaban	1	50	3	1
53	LMWH	0	25	3	1
54	fondaparinux	44	784	1	1
54	LMWH	65	796	1	1
55	rivaroxaban	14	60	1	1
55	LMWH	31	70	1	1
56	rivaroxaban	61	965	1	1
56	LMWH	86	959	1	1
57	betrixaban	9	65	1	1
57	LMWH	4	40	1	1
58	rivaroxaban	8	96	3	1
58	LMWH	17	99	3	1
59	rivaroxaban	1	89	2	1
59	LMWH	4	89	2	1
60	rivaroxaban	1	60	1	1
60	LMWH	3	60	1	1
61	LMWH	21	76	3	1
61	apixaban	12	83	3	1

62	rivaroxaban	3	15	2	1
62	LMWH	4	15	2	1
63	rivaroxaban	4	75	3	1
63	LMWH	3	70	3	1
64	fondaparinux	6	84	1	1
64	LMWH	5	83	1	1
65	LMWH	6	45	3	1
65	rivaroxaban	7	45	3	1
66	LMWH	6	43	3	1
66	rivaroxaban	1	43	3	1
67	rivaroxaban	3	102	2	1
67	LMWH	14	112	2	1
67	aspirin	18	110	2	1
