# Supplementary material for: Efficacy of 11 anticoagulants for the prevention of venous thromboembolism after total hip or knee arthroplasty: A systematic review and network meta-analysis
Source: Medicine (Baltimore). 2023 Jan 13;102(2):e32635. doi: 10.1097/MD.0000000000032635 (PMC9839234; doi:10.1097/MD.0000000000032635)
Supplement: Supplementary file 5 [file medi-102-e32635-s005.pdf]

Supplemental Table 4 PE outcome’s data format that be used as input to CINeMA. Rob and indirectness can take either 1, 2, and 3 values for low, moderate, and high risk of bias or level of indirectness.

NO.	study	r	n	rob	indirectness
1	LMWH	3	398	1	1
1	aspirin	0	380	1	1
2	rivaroxaban	4	902	1	1
2	aspirin	3	902	1	1
3	rivaroxaban	4	815	1	1
3	aspirin	4	805	1	1
4	rivaroxaban	0	42	2	1
4	LMWH	0	42	2	1
5	rivaroxaban	0	114	2	1
5	LMWH	0	114	2	1
6	fondaparinux	1	517	1	1
6	LMWH	4	517	1	1
7	rivaroxaban	0	60	2	1
7	LMWH	0	60	2	1
8	dabigatran	0	297	1	1
8	LMWH	0	300	1	1
9	dabigatran	0	675	1	1
9	LMWH	1	685	1	1
10	dabigatran	5	1137	1	1
10	LMWH	3	1142	1	1
11	rivaroxaban	0	59	2	1
11	LMWH	0	107	2	1
12	rivaroxaban	4	1595	1	1
12	LMWH	1	1558	1	1
13	darexaban	0	120	1	1
13	LMWH	0	127	1	1
14	dabigatran	1	1001	1	1
14	LMWH	2	992	1	1
15	dabigatran	1	1001	1	1
15	LMWH	2	992	1	1
16	warfarin	0	122	1	1
16	LMWH	0	108	1	1
17	edoxaban	0	299	1	1
17	LMWH	0	295	1	1
18	edoxaban	0	255	1	1
18	LMWH	0	248	1	1
19	dabigatran	6	604	1	1
19	LMWH	5	643	1	1
20	LMWH	1	813	1	1

20	UFH	1	815	1	1
21	fondaparinux	1	277	3	1
21	LMWH	5	297	3	1
22	rivaroxaban	1	864	1	1
22	LMWH	4	869	1	1
23	LMWH	1	125	1	1
23	UFH	2	134	1	1
24	rivaroxaban	2	350	1	1
24	LMWH	1	351	1	1
25	fondaparinux	2	1129	1	1
25	LMWH	2	1123	1	1
26	apixaban	0	105	1	1
26	LMWH	2	109	1	1
26	warfarin	0	109	1	1
27	rivaroxaban	0	824	1	1
27	LMWH	4	878	1	1
28	fondaparinux	1	360	3	1
28	LMWH	0	223	3	1
28	UFH	0	72	3	1
29	fondaparinux	0	261	3	1
29	LMWH	0	148	3	1
29	UFH	0	32	3	1
30	LMWH	0	45	1	1
30	dabigatran	0	45	1	1
31	rivaroxaban	1	50	2	1
31	LMWH	2	50	2	1
32	rivaroxaban	0	48	2	1
32	LMWH	0	36	2	1
33	rivaroxaban	0	80	2	1
33	LMWH	0	80	2	1
34	LMWH	0	50	3	1
34	UFH	0	50	3	1
35	fondaparinux	5	1126	1	1
35	LMWH	1	1128	1	1
36	rivaroxaban	0	60	1	1
36	LMWH	0	70	1	1
37	rivaroxaban	5	1526	1	1
37	LMWH	8	1508	1	1
38	betrixaban	1	65	1	1
38	LMWH	0	40	1	1
39	LMWH	0	76	3	1
39	apixaban	0	83	3	1
40	rivaroxaban	0	15	2	1

40	LMWH	0	15	2	1
41	fondaparinux	0	84	1	1
41	LMWH	0	83	1	1
42	rivaroxaban	0	102	2	1
42	LMWH	0	112	2	1
42	aspirin	0	110	2	1
