# Supplementary material for: Efficacy of 11 anticoagulants for the prevention of venous thromboembolism after total hip or knee arthroplasty: A systematic review and network meta-analysis
Source: Medicine (Baltimore). 2023 Jan 13;102(2):e32635. doi: 10.1097/MD.0000000000032635 (PMC9839234; doi:10.1097/MD.0000000000032635)
Supplement: Supplementary file 7 [file medi-102-e32635-s007.pdf]

Supplemental Table 6 Final output of CINeMA for the network of anticoagulants for prevention of PE.

Comparison	Number of studies	Within-study bias	Reporting bias	Indirectness	Imprecision	Heterogeneity	Incoherence	Confidence rating	Reason(s) for downgrading
LMWH:UFH	5	Some concerns	Low risk	No concern	Major concerns	No concern	No concern	Very low	Within-study bias, Serious Imprecision
apixaban:LMWH	2	Some concerns	Low risk	No concern	Major concerns	No concern	No concern	Very low	Within-study bias, Serious Imprecision
aspirin:LMWH	2	No concern	Low risk	No concern	Major concerns	No concern	No concern	Low	Serious Imprecision
betrixaban:LMWH	1	No concern	Low risk	No concern	Major concerns	No concern	No concern	Low	Serious Imprecision
dabigatran:LMWH	7	No concern	Low risk	No concern	Major concerns	No concern	No concern	Low	Serious Imprecision
darexaban:LMWH	1	No concern	Low risk	No concern	Major concerns	No concern	No concern	Low	Serious Imprecision
edoxaban:LMWH	2	No concern	Low risk	No concern	Major concerns	No concern	No concern	Low	Serious Imprecision
fondaparinux:LMWH	7	Some concerns	Low risk	No concern	Major concerns	No concern	No concern	Very low	Within-study bias, Serious Imprecision
LMWH:rivaroxaban	15	No concern	Low risk	No concern	Major concerns	No concern	No concern	Low	Serious Imprecision
LMWH:warfarin	2	No concern	Low risk	No concern	Major concerns	No concern	No concern	Low	Serious Imprecision
fondaparinux:UFH	2	Some concerns	Low risk	No concern	Major concerns	No concern	No concern	Very low	Within-study bias, Serious Imprecision
apixaban:warfarin	1	No concern	Low risk	No concern	Major concerns	No concern	No concern	Low	Serious Imprecision
aspirin:rivaroxaban	3	No concern	Low risk	No concern	Major concerns	No concern	No concern	Low	Serious Imprecision
apixaban:UFH	0	Some concerns	Low risk	No concern	Major concerns	No concern	No concern	Very low	Within-study bias, Serious Imprecision
aspirin:UFH	0	No concern	Low risk	No concern	Major concerns	No concern	No concern	Low	Serious Imprecision
betrixaban:UFH	0	No concern	Low risk	No concern	Major concerns	No concern	No concern	Low	Serious Imprecision
dabigatran:UFH	0	No concern	Low risk	No concern	Major concerns	No concern	No concern	Low	Serious Imprecision
darexaban:UFH	0	No concern	Low risk	No concern	Major concerns	No concern	No concern	Low	Serious Imprecision
edoxaban:UFH	0	No concern	Low risk	No concern	Major concerns	No concern	No concern	Low	Serious Imprecision
rivaroxaban:UFH	0	Some concerns	Low risk	No concern	Major concerns	No concern	No concern	Very low	Within-study bias, Serious Imprecision
UFH:warfarin	0	No concern	Low risk	No concern	Major concerns	No concern	No concern	Low	Serious Imprecision
apixaban:aspirin	0	No concern	Low risk	No concern	Major concerns	No concern	No concern	Low	Serious Imprecision
apixaban:betrixaban	0	No concern	Low risk	No concern	Major concerns	No concern	No concern	Low	Serious Imprecision
apixaban:dabigatran	0	No concern	Low risk	No concern	Major concerns	No concern	No concern	Low	Serious Imprecision
apixaban:darexaban	0	No concern	Low risk	No concern	Major concerns	No concern	No concern	Low	Serious Imprecision
apixaban:edoxaban	0	No concern	Low risk	No concern	Major concerns	No concern	No concern	Low	Serious Imprecision
apixaban:fondaparinux	0	Some concerns	Low risk	No concern	Major concerns	No concern	No concern	Very low	Within-study bias, Serious Imprecision
apixaban:rivaroxaban	0	No concern	Low risk	No concern	Major concerns	No concern	No concern	Low	Serious Imprecision
aspirin:betrixaban	0	No concern	Low risk	No concern	Major concerns	No concern	No concern	Low	Serious Imprecision
aspirin:dabigatran	0	No concern	Low risk	No concern	Major concerns	No concern	No concern	Low	Serious Imprecision
aspirin:darexaban	0	No concern	Low risk	No concern	Major concerns	No concern	No concern	Low	Serious Imprecision
aspirin:edoxaban	0	No concern	Low risk	No concern	Major concerns	No concern	No concern	Low	Serious Imprecision
aspirin:fondaparinux	0	No concern	Low risk	No concern	Major concerns	No concern	No concern	Low	Serious Imprecision
aspirin:warfarin	0	No concern	Low risk	No concern	Major concerns	No concern	No concern	Low	Serious Imprecision
betrixaban:dabigatran	0	No concern	Low risk	No concern	Major concerns	No concern	No concern	Low	Serious Imprecision
betrixaban:darexaban	0	No concern	Low risk	No concern	Major concerns	No concern	No concern	Low	Serious Imprecision
betrixaban:edoxaban	0	No concern	Low risk	No concern	Major concerns	No concern	No concern	Low	Serious Imprecision
betrixaban:fondaparinux	0	No concern	Low risk	No concern	Major concerns	No concern	No concern	Low	Serious Imprecision
betrixaban:rivaroxaban	0	No concern	Low risk	No concern	Major concerns	No concern	No concern	Low	Serious Imprecision
betrixaban:warfarin	0	No concern	Low risk	No concern	Major concerns	No concern	No concern	Low	Serious Imprecision
dabigatran:darexaban	0	No concern	Low risk	No concern	Major concerns	No concern	No concern	Low	Serious Imprecision
dabigatran:edoxaban	0	No concern	Low risk	No concern	Major concerns	No concern	No concern	Low	Serious Imprecision
dabigatran:fondaparinux	0	No concern	Low risk	No concern	Major concerns	No concern	No concern	Low	Serious Imprecision
dabigatran:rivaroxaban	0	No concern	Low risk	No concern	Major concerns	No concern	No concern	Low	Serious Imprecision
dabigatran:warfarin	0	No concern	Low risk	No concern	Major concerns	No concern	No concern	Low	Serious Imprecision
darexaban:edoxaban	0	No concern	Low risk	No concern	Major concerns	No concern	No concern	Low	Serious Imprecision
darexaban:fondaparinux	0	No concern	Low risk	No concern	Major concerns	No concern	No concern	Low	Serious Imprecision
darexaban:rivaroxaban	0	No concern	Low risk	No concern	Major concerns	No concern	No concern	Low	Serious Imprecision
darexaban:warfarin	0	No concern	Low risk	No concern	Major concerns	No concern	No concern	Low	Serious Imprecision
edoxaban:fondaparinux	0	No concern	Low risk	No concern	Major concerns	No concern	No concern	Low	Serious Imprecision
edoxaban:rivaroxaban	0	No concern	Low risk	No concern	Major concerns	No concern	No concern	Low	Serious Imprecision
edoxaban:warfarin	0	No concern	Low risk	No concern	Major concerns	No concern	No concern	Low	Serious Imprecision
fondaparinux:rivaroxaban	0	No concern	Low risk	No concern	Major concerns	No concern	No concern	Low	Serious Imprecision
fondaparinux:warfarin	0	No concern	Low risk	No concern	Major concerns	No concern	No concern	Low	Serious Imprecision
rivaroxaban:warfarin	0	No concern	Low risk	No concern	Major concerns	No concern	No concern	Low	Serious Imprecision
