# Supplementary material for: Efficacy of 11 anticoagulants for the prevention of venous thromboembolism after total hip or knee arthroplasty: A systematic review and network meta-analysis
Source: Medicine (Baltimore). 2023 Jan 13;102(2):e32635. doi: 10.1097/MD.0000000000032635 (PMC9839234; doi:10.1097/MD.0000000000032635)
Supplement: Supplementary file 6 [file medi-102-e32635-s006.pdf]

Supplemental Table 5 Final output of CINeMA for the network of anticoagulants for prevention of DVT.

Comparison	Number of studies	Within-study bias	Reporting bias	Indirectness	Imprecision	Heterogeneity	Incoherence	Confidence rating	Reason(s) for downgrading
LMWH:UFH	5	No concern	Low risk	No concern	Some concerns	Some concerns	No concern	Low	Imprecision, Heterogeneity
apixaban:LMWH	3	Major concerns	Low risk	No concern	No concern	No concern	No concern	Low	Serious Within-study bias
aspirin:LMWH	3	Some concerns	Low risk	No concern	Major concerns	No concern	No concern	Very low	Within-study bias, Serious Imprecision
betrixaban:LMWH	1	No concern	Low risk	No concern	Major concerns	No concern	No concern	Low	Serious Imprecision
dabigatran:LMWH	9	No concern	Low risk	No concern	No concern	Major concerns	No concern	Low	Serious Heterogeneity
darexaban:LMWH	4	Some concerns	Low risk	No concern	Some concerns	Some concerns	No concern	Very low	Within-study bias, Imprecision, Heterogeneity
edoxaban:LMWH	3	No concern	Low risk	No concern	No concern	No concern	No concern	High	
fondaparinux:LMWH	9	Some concerns	Low risk	No concern	No concern	Some concerns	No concern	Low	Within-study bias, Heterogeneity
LMWH:rivaroxaban	27	No concern	Low risk	No concern	No concern	No concern	No concern	High	
LMWH:warfarin	3	No concern	Low risk	No concern	No concern	Some concerns	No concern	Moderate	Heterogeneity
fondaparinux:UFH	2	Some concerns	Low risk	No concern	No concern	No concern	No concern	Moderate	Within-study bias
apixaban:warfarin	1	Some concerns	Low risk	No concern	No concern	No concern	No concern	Moderate	Within-study bias
aspirin:rivaroxaban	5	Some concerns	Low risk	No concern	No concern	Some concerns	No concern	Low	Within-study bias, Heterogeneity
apixaban:UFH	0	Some concerns	Low risk	No concern	No concern	No concern	No concern	Moderate	Within-study bias
aspirin:UFH	0	Some concerns	Low risk	No concern	Major concerns	No concern	No concern	Very low	Within-study bias, Serious Imprecision
betrixaban:UFH	0	No concern	Low risk	No concern	Major concerns	No concern	No concern	Low	Serious Imprecision
dabigatran:UFH	0	No concern	Low risk	No concern	Some concerns	Some concerns	No concern	Low	Imprecision, Heterogeneity
darexaban:UFH	0	Some concerns	Low risk	No concern	No concern	Some concerns	No concern	Low	Within-study bias, Heterogeneity
edoxaban:UFH	0	No concern	Low risk	No concern	No concern	No concern	No concern	High	
rivaroxaban:UFH	0	No concern	Low risk	No concern	No concern	No concern	No concern	High	
UFH:warfarin	0	No concern	Low risk	No concern	No concern	Major concerns	No concern	Low	Serious Heterogeneity
apixaban:aspirin	0	Some concerns	Low risk	No concern	No concern	No concern	No concern	Moderate	Within-study bias
apixaban:betrixaban	0	Some concerns	Low risk	No concern	No concern	Some concerns	No concern	Low	Within-study bias, Heterogeneity
apixaban:dabigatran	0	Some concerns	Low risk	No concern	No concern	No concern	No concern	Moderate	Within-study bias
apixaban:darexaban	0	Some concerns	Low risk	No concern	Some concerns	Some concerns	No concern	Very low	Within-study bias, Imprecision, Heterogeneity
apixaban:edoxaban	0	Some concerns	Low risk	No concern	Major concerns	No concern	No concern	Very low	Within-study bias, Serious Imprecision
apixaban:fondaparinux	0	Some concerns	Low risk	No concern	Some concerns	Some concerns	No concern	Very low	Within-study bias, Imprecision, Heterogeneity
apixaban:rivaroxaban	0	Some concerns	Low risk	No concern	Some concerns	Some concerns	No concern	Very low	Within-study bias, Imprecision, Heterogeneity
aspirin:betrixaban	0	Some concerns	Low risk	No concern	Major concerns	No concern	No concern	Very low	Within-study bias, Serious Imprecision
aspirin:dabigatran	0	Some concerns	Low risk	No concern	Major concerns	No concern	No concern	Very low	Within-study bias, Serious Imprecision
aspirin:darexaban	0	Some concerns	Low risk	No concern	Some concerns	Some concerns	No concern	Very low	Within-study bias, Imprecision, Heterogeneity
aspirin:edoxaban	0	Some concerns	Low risk	No concern	No concern	No concern	No concern	Moderate	Within-study bias
aspirin:fondaparinux	0	Some concerns	Low risk	No concern	No concern	Some concerns	No concern	Low	Within-study bias, Heterogeneity
aspirin:warfarin	0	Some concerns	Low risk	No concern	No concern	Some concerns	No concern	Low	Within-study bias, Heterogeneity
betrixaban:dabigatran	0	No concern	Low risk	No concern	Major concerns	No concern	No concern	Low	Serious Imprecision
betrixaban:darexaban	0	No concern	Low risk	No concern	Major concerns	No concern	No concern	Low	Serious Imprecision
betrixaban:edoxaban	0	No concern	Low risk	No concern	No concern	Some concerns	No concern	Moderate	Heterogeneity
betrixaban:fondaparinux	0	No concern	Low risk	No concern	Some concerns	Some concerns	No concern	Low	Imprecision, Heterogeneity
betrixaban:rivaroxaban	0	No concern	Low risk	No concern	Major concerns	No concern	No concern	Low	Serious Imprecision
betrixaban:warfarin	0	No concern	Low risk	No concern	Major concerns	No concern	No concern	Low	Serious Imprecision
dabigatran:darexaban	0	No concern	Low risk	No concern	Some concerns	Some concerns	No concern	Low	Imprecision, Heterogeneity
dabigatran:edoxaban	0	No concern	Low risk	No concern	No concern	No concern	No concern	High	
dabigatran:fondaparinux	0	No concern	Low risk	No concern	No concern	Major concerns	No concern	Low	Serious Heterogeneity
dabigatran:rivaroxaban	0	No concern	Low risk	No concern	No concern	Some concerns	No concern	Moderate	Heterogeneity
dabigatran:warfarin	0	No concern	Low risk	No concern	No concern	No concern	No concern	High	
darexaban:edoxaban	0	No concern	Low risk	No concern	Some concerns	Some concerns	No concern	Low	Imprecision, Heterogeneity
darexaban:fondaparinux	0	Some concerns	Low risk	No concern	Major concerns	No concern	No concern	Very low	Within-study bias, Serious Imprecision
darexaban:rivaroxaban	0	Some concerns	Low risk	No concern	Major concerns	No concern	No concern	Very low	Within-study bias, Serious Imprecision
darexaban:warfarin	0	Some concerns	Low risk	No concern	No concern	No concern	No concern	Moderate	Within-study bias
edoxaban:fondaparinux	0	No concern	Low risk	No concern	Some concerns	Some concerns	No concern	Low	Imprecision, Heterogeneity
edoxaban:rivaroxaban	0	No concern	Low risk	No concern	Some concerns	Some concerns	No concern	Low	Imprecision, Heterogeneity
edoxaban:warfarin	0	No concern	Low risk	No concern	No concern	No concern	No concern	High	
fondaparinux:rivaroxaban	0	Some concerns	Low risk	No concern	Some concerns	Some concerns	No concern	Very low	Within-study bias, Imprecision, Heterogeneity
fondaparinux:warfarin	0	Some concerns	Low risk	No concern	No concern	No concern	No concern	Moderate	Within-study bias
rivaroxaban:warfarin	0	No concern	Low risk	No concern	No concern	No concern	No concern	High	
